# Dialectical behaviour therapy for posttraumatic stress disorder related to childhood sexual abuse: a pilot study in an outpatient treatment setting

**DOI:** 10.1080/20008198.2018.1423832

**Published:** 2018-01-19

**Authors:** Regina Steil, Clara Dittmann, Meike Müller-Engelmann, Anne Dyer, Anne-Marie Maasch, Kathlen Priebe

**Affiliations:** ^a^ Department of Clinical Psychology and Intervention, Institute of Psychology, Goethe University, Frankfurt Main, Germany; ^b^ Central Institute of Mental Health, Medical Faculty Mannheim/Heidelberg University, Mannheim, Germany; ^c^ Private Practice, Bad Homburg, Germany; ^d^ Department of Psychotherapy and Somatopsychology, Humboldt University, Berlin, Germany

**Keywords:** Borderline personality disorder, childhood sexual abuse, trauma, cognitive behaviour therapy, psychotherapy outcome research, feasibility, trastorno borderline de personalidad, abuso sexual infantil, trauma, terapia cognitivo conductual, investigación de resultados psicoterapéuticos, aplicabilidad, 边缘性人格障碍, 童年性虐待, 创伤, 认知行为治疗, 心理治疗结果研究, 可行性, • Safety, acceptance and effects of outpatient Dialectical Behaviour Therapy for PTSD were evaluated.• Pre–post and pre–follow-up treatment effects of DBT-PTSD were found to be large for PTSD symptoms, Borderline symptoms and depression.• 79% of the patients showed remission in PTSD.• DBT-PTSD showed good acceptance with normal dropout rate of 19%.

## Abstract

**Background**: Dialectical behaviour therapy for posttraumatic stress disorder (DBT-PTSD), which is tailored to treat adults with PTSD and co-occurring emotion regulation difficulties, has already demonstrated its efficacy, acceptance and safety in an inpatient treatment setting. It combines elements of DBT with trauma-focused cognitive behavioural interventions.

**Objective**: To investigate the feasibility, acceptance and safety of DBT-PTSD in an outpatient treatment setting by therapists who were novice to the treatment, we treated 21 female patients suffering from PTSD following childhood sexual abuse (CSA) plus difficulties in emotion regulation in an uncontrolled clinical trial.

**Method**: The Clinician Administered PTSD Symptom Scale (CAPS), the Davidson Trauma Scale (DTS), the Borderline Section of the International Personality Disorder Examination (IPDE) and the Borderline Symptom List (BSL-23) were used as primary outcomes. For secondary outcomes, depression and dissociation were assessed. Assessments were administered at pretreatment, post-treatment and six-week follow-up.

**Results**: Improvement was significant for PTSD as well as for borderline personality symptomatology, with large pretreatment to follow-up effect sizes for completers based on the CAPS (Cohens *d* = 1.30), DTS (*d* = 1.50), IPDE (*d* = 1.60) and BSL-23 (*d* = 1.20).

**Conclusion**: The outcome suggests that outpatient DBT-PTSD can safely be used to reduce PTSD symptoms and comorbid psychopathology in adults who have experienced CSA.

## Abstract (es)

**Contexto**: La terapia cognitiva dialéctica (TCD) para el Trastorno de Estrés Postraumático (DBT-PTSD), echa a medida para tratar personas adultas con TEPT así como dificultades de regulación afectiva simultaneas, ha demostrado ya su eficacia, aceptación y seguridad en el marco de un programa de tratamiento con pacientes ingresados. Esta terapia combina elementos de DBT con intervenciones cognitivo conductuales focalizadas en el trauma.

**Objetivos**: Investigar la viabilidad y aplicabilidad y seguridad de un programa de tratamiento con TCD- TEPT en un grupo de pacientes en régimen de tratamiento ambulatorio llevado a cabo por terapeutas noveles. Se trataron a 21 pacientes femeninas, en un ensayo clínico no controlado que sufrían de TEPT consecutivo a abuso sexual infantil (ASI) así como dificultades en la regulación emocional.

**Método**: Se aplicaron, cara a la obtención de resultados válidos, los siguientes instrumentos: las Escalas de Síntomas de TEPT para ser administradas por un clínico (CAPS), la escala de trauma de Davidson (DTS), la sección para pacientes borderline de la versión internacional del Examen del trastorno de personalidad (IPDE) y, finalmente, la lista de síntomas borderline (BSL-23). Para la obtención de resultados de segundo orden, se determino la presencia de depresión y disociación. La evaluaciones se hicieron antes de la aplicación del tratamiento, después de la aplicación del mismo y en un seguimiento realizado 6 semanas después.

**Resultados**: La mejoría fue significativa tanto para el TEPT como para sintomatología de personalidad borderline, con importantes magnitudes de efecto para aquellos que completaron las escalas CAPS (Cohens *d *= 1.30), DTS (*d* = 1.50), IPDE (*d* = 1.60) y BSL-23 (*d* = 1.20).

**Conclusiones**: El resultado sugiere que el tratamiento ambulatorio TCD – TEPT puede ser aplicado con seguridad para reducir los síntomas de TEPT así como la psicopatología comórbida, en pacientes que experimentaron abuso sexual infantil.

## Abstract (zh)

背景：创伤后应激障碍的辩证行为疗法（DBT-PTSD） 是为了治疗同时患有 PTSD 和情绪调控困难的成年人而制订的。该疗法同时结合了 DBT 的元素和创伤中心的认知行为干预，已经在住院病人中验证了它的效果、接受度和安全性。

目标：为了在门诊病人治疗情景中考察 DBT-PTSD 的可行性、接受度和安全性，我们让新手治疗师在一个无控制临床试验中治疗了21位患有童年性虐待（CSA）后的 PTSD并有情绪调控问题的女性病人。

方法：使用《临床医师用PTSD症状量表》（CAPS），《戴维森创伤量表》（DTS），《国际人格障碍检验的边缘性分类》（IPDE）和《边缘性症状清单》（BSL-23）主要结果。测量了抑郁和分离作为次要结果。评估在治疗前，治疗后和6周追踪期进行。

结果：在完成被试中，从治疗前到追踪期PTSD 和边缘性人格障碍症状的改善是显著的，效应量分别为：CAPS (Cohens d = 1.30), DTS (d = 1.50), IPDE (d = 1.60) 和BSL-23 (d = 1.20)。

结论：结果提示 门诊病人DBT-PTSD中可以安全地在经历过 CSA 的成人中使用来减少 PTSD症状和共病。

## 1. Introduction

Early traumatic experiences, such as childhood sexual abuse (CSA), are associated with lifetime consequences, such as severe mental or somatic disorders (Gilbert et al., 2009; Irish, Kobayashi, & Delahanty, 2010). Victims often develop posttraumatic stress disorder (PTSD) and pervasive problems or mental disorders associated with difficulties in emotion regulation, such as borderline personality disorder (BPD) (Maniglio, 2009).

**Figure 1.**
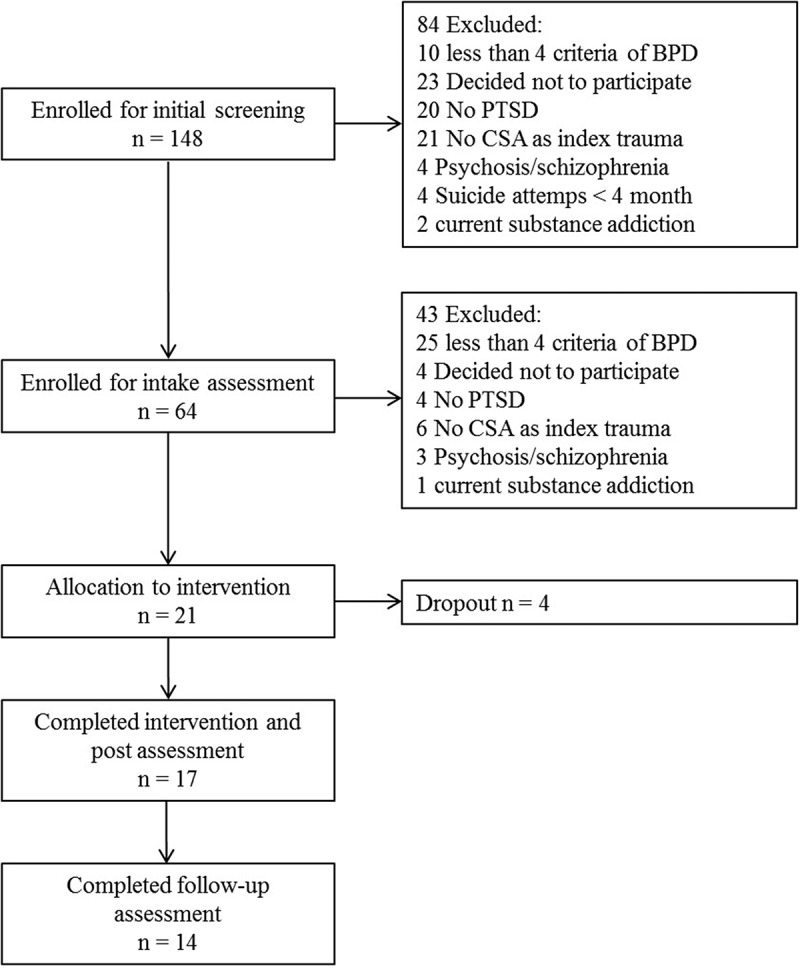
Patient flow chart.

Meta-analyses (e.g. Watts et al., 2013) recommend trauma-focused cognitive-behavioural therapy for effective PTSD treatment. However, treatments for CSA-related PTSD are scarce and are less effective than those for PTSD following other traumas (Ehring et al., 2014), especially in patients with more complex symptomatology (Dorrepaal et al., 2014). There is a lack of established, evidenced-based treatments tailored to the needs of these patients (Bohus et al., 2013; Priebe et al., 2012).

Steil and colleagues (Steil, Dyer, Priebe, Kleindienst, & Bohus, 2011) developed the following inpatient treatment specifically tailored to the needs of patients suffering from PTSD following CSA plus difficulties in emotion regulation: Dialectical Behaviour Therapy for Posttraumatic Stress Disorder (DBT-PTSD). In the two trials completed thus far, inpatient DBT-PTSD has demonstrated efficacy (Bohus et al., 2013; Steil et al., 2011). Compared to the usual treatment, DBT-PTSD was significantly superior in reducing PTSD symptoms, with large between-group effect sizes. Neither a diagnosis of BPD nor BPD symptom severity was significantly related to treatment outcome (Bohus et al., 2013).

Inpatient treatment, however, is expensive and can only be offered to a small number of patients. Therefore, we carried out a non-controlled pilot trial investigating the safety, the acceptability and the effects of a 24-session course of outpatient DBT-PTSD in patients suffering from CSA-related PTSD plus problems in emotion regulation, treated by therapists who were novice to the treatment. We expected outpatient DBT-PTSD to significantly reduce PTSD and BPD symptom severity as well as the secondary outcomes of depression and dissociation.

## 2. Method

### 2.1. Intervention

DBT-PTSD is a modular treatment programme. It is based on the principles and methods of Dialectical Behaviour Therapy (DBT; Linehan, 1993) and integrates trauma-focused cognitive and exposure-based interventions (Bohus et al., 2013; Steil et al., 2011). The DBT-PTSD programme follows the DBT hierarchy of treatment targets, which prioritizes life-threatening behaviours, such as suicide attempts, and treatment-interfering behaviours, such as dissociation, over addressing problems reducing quality of life, such as sexual problems. Outpatient DBT-PTSD is structured into the following five stages: (1) Anamnestic information is collected, psychoeducation is given and a treatment contract is signed; (2) Treatment aims are defined, an introduction to mindfulness is given, concerns regarding exposure are addressed and the therapist and patient develop an individual model of PTSD development and maintenance. Furthermore, distress tolerance skills from DBT are implemented to address problematic behaviour; (3) Typical strategies to escape distressing trauma-related emotions on a behavioural, cognitive and emotional level (such as self-harm, dissociation or feelings of guilt) are identified and addressed; (4) Exposure-based techniques are applied. The exposure protocol allows the patient to control the intensity of memory activation and balances the vividness of trauma memories with the awareness of being in the (non-dangerous) present, by using skills during exposure sessions and exposure homework (skills-assisted exposure); (5) Treatment focuses on radical acceptance of trauma-related facts and on relevant psychosocial problems, including work and partnership. An additional booster session is applied six weeks post-treatment.

For the present study, nine clinical psychologists who were novice to the treatment (all but one were still in training and had 1–2 years of clinical experience) were trained with a four-day workshop on DBT-PTSD; furthermore, they completed six one-day workshops in basic DBT. They administered up to 24 weekly sessions with a maximum of 40 hours of individual treatment, which were flexible in length and could last between 50 and 120 minutes. Treatment adherence was secured by regular weekly supervision by the first author, with an average of 90 minutes of group supervision per week.

### 2.2. Procedure

Patients were recruited from our specialized PTSD outpatient centre at the Goethe University of Frankfurt. Those who seemed eligible for the study were contacted by phone, informed about the study and invited to an initial screening session. Those who seemed suitable afterwards were invited to two further pretreatment assessment sessions, where inclusion and exclusion criteria were checked.

The inclusion criteria included a DSM-IV (American Psychiatric Association, 1994) diagnosis of PTSD after CSA before the age of 18 as the current most distressing, traumatic event (index trauma), determined by the Clinician-Administered PTSD Scale (CAPS; Schnyder & Moergeli, 2002). Furthermore, participants had to fulfil a minimum of four criteria of BPD as measured by the International Personality Disorder Examination (IPDE; Loranger et al., 1994). Patients had to be older than 17 years and had to give informed consent.

The exclusion criteria included a current clinical diagnosis of substance dependence, a lifetime diagnosis of schizophrenia or bipolar I disorder, all according to the Structured Clinical Interview for the DSM-IV (SCID I; Wittchen, Zaudig, & Fydrich, 1997), mental retardation, a body mass index (BMI) < 16.5 and suicide attempts or severe, life-threatening self-harm within the last 18 weeks, according to the Severe Behaviour Dyscontrol Interview (SBD-I; Bohus & Borgmann, 2012).

Figure 1 shows the patient flow. Twenty-one participants met the eligibility criteria, gave informed consent and began DBT-PTSD treatment. Outcomes were assessed at pretreatment, post-treatment and at six-week follow-up by clinical interviews administered by trained independent raters (clinical psychologists) and via self-report measures. Seventeen participants completed the intervention and post-treatment assessment. Fourteen of them also completed the follow-up assessment six weeks later (study completer group). This study was carried out in accordance with the recommendations of the ethics committee of the Goethe-University Frankfurt. All subjects gave written informed consent in accordance with the Declaration of Helsinki.

### 2.3. Measures

#### 2.3.1. Primary outcome

The Clinician-Administered PTSD Scale for DSM-IV (CAPS; Blake et al., 1995; Schnyder & Moergeli, 2002) measures the frequency and intensity of 17 PTSD symptoms over the past four weeks. This interview was conducted twice, first focusing on symptoms regarding the three most-distressing traumatic events (multiple traumas) and, second, regarding the most distressing single event (index trauma), which had to be CSA. We used the recommended scoring rule requiring a frequency score of ‘one’ and an intensity score of ‘two’ to consider a symptom to be present (Weathers, Ruscio, & Keane, 1999). A severity score is built by summing up the frequency and intensity scores for the 17 symptoms (range 0–136). Cronbach’s α was found to be good, with α = .88 for the total severity score (Schnyder & Moergeli, 2002).

The Davidson Trauma Scale (DTS; Davidson et al., 1997) is a 17-item self-rating scale that assesses the frequency and severity of 17 PTSD symptoms within the past week on a five-point Likert scale, ranging from zero to four. The sum score ranges from zero to 136. Cronbach’s α was found to be α = .99 (Davidson et al., 1997), indicating very good reliability.

The International Personality Disorder Examination (IPDE; Loranger et al., 1994) is a semi-structured interview designed to assess personality disorders according to ICD-10. We used the Borderline section, where 15 questions are addressed to identify how many of the nine criteria of BPD are currently (last five years including the last month) fulfilled. If five or more criteria are met, a BPD diagnosis is confirmed. A dimensional score can be built using a three-point Likert scale for each question, ranging from zero to two (range: 0–30). The IPDE shows good interrater-reliability, with κ = .79 (Loranger et al., 1994).

The Borderline Symptom List (BSL-23; Bohus et al., 2009) is a 23-item short form that uses a self-rating scale to assess the severity of BPD symptoms on a five-point Likert scale. In addition to the sum score (range: 0–92), global functioning and dysfunctional behaviour can be assessed separately. Cronbach’s α was found to be α = .94–.97 (Bohus et al., 2009).

#### 2.3.2. Secondary outcome

The Beck Depression Inventory (BDI-II; Beck, Steer, & Brown, 1996; Hautzinger, Keller, & Kühner, 2006) is a 21-item questionnaire that measures depressive symptoms over the last two weeks. Answers are given on a four-point Likert scale with at least four possible response options indicating increasing intensity. Cronbach’s α was found to be α = .91 (Hautzinger et al., 2006).

The Dissociative Experience Scale (FDS; German adaptation by Spitzer, Stieglitz, & Freyberger, 2005) is a self-rated screening tool for dissociative symptoms and consists of 20 items regarding a variety of dissociative symptoms. The frequency of the symptoms is assessed by a percentage scale from 0 (never) to 100 (always). Cronbach’s α was found to be α = .94 (Spitzer, Mestel, Klingelhöfer, Gänsicke, & Freyberger, 2004).

An adapted version of the Severe Behaviour Dyscontrol Interview (SBD-I; Bohus & Borgmann, 2012) was used at pretreatment and follow-up to collect participants’ history of self-harm behaviours and suicide attempts during the 18 weeks prior to study entry as well as during the past four weeks.

### 2.4. Statistical analysis

Single missing items were substituted with multiple imputation, in which one complete dataset is calculated. Imputation in SPSS is based on a Markov chain Monte Carlo algorithm (MCMC; Little & Rubin, 2002). We used hierarchical linear models (two levels; observations [ratings] were clustered in participants) to define the changes in symptoms of PTSD and BPD over time (from baseline to post-treatment and follow-up). Based on the given data structure, we found these models to analyse the effects of the intervention on PTSD and BPD symptoms best because they allowed the inclusion of the baseline values of all starters and the post-treatment and follow-up values of completers in the case of dropouts. The significance level was set at *p* < .05 (two-tailed). Cohen’s *d* (Cohen, 1977) was used to calculate treatment effect sizes. We calculated effect sizes for the intention-to-treat sample (ITT), including all patients who completed the pretreatment assessment (*N* = 21) as well as for study completers (*N* = 14). We decided to use last-observation-carried-forward (LOCF) for dropouts in ITT analyses. The effect sizes were defined as small (*d *> 0.20), medium (*d *> 0.50) and large (*d *> 0.80) (see Cohen, 1977). The CAPS effect-sizes were calculated for symptoms in relation to multiple traumas as well as for symptoms in relation to the index trauma (Priebe et al., submitted). Statistics were calculated using SPSS®, Version 22. Remission with regard to PTSD was defined as no longer meeting DSM-IV criteria according to the CAPS interview.

## 3. Results

### 3.1. Participant characteristics

ITT consisted of 21 women with an average age of 34.05 years (*SD* = 9.34; range 19–50 years). Seven participants (33.3%) held a high school degree, 11 (47.4%) had finished secondary school, one (4.8%) was without a school-leaving qualification and two (9.5%) were still in vocational education. Fourteen (67%) were living in a stable partnership, six (24%) had children.

The average age at the onset of the index trauma was 11.29 years (*SD* = 4.19), the CSA lasted 6.02 years (72.24 months; *SD *= 110.18) on average. Regarding CSA, six participants (29%) reported a single event, five (24%) were abused up to 20 times and eight (38%) more than 20 times; two participants did not report frequency. In nine cases (43%), the perpetrator was living in the same household as a parent or sibling; in eight cases (33%), the perpetrator was a more distant acquaintance, such as a neighbour; and in four cases (19%), the perpetrator was a stranger. Fifteen participants (71%) reported sexual as well as physical childhood abuse. On average, 3.5 different trauma clusters (e.g. CSA by perpetrator x and later CSA by perpetrator y) were reported by the participants.

Prior to treatment, the average duration of PTSD was 14.47 years (*SD *= 12.91; range 2 months to 38 years). The average number of DSM-IV axis-I disorders in addition to PTSD was 2.95 (*SD *= 1.19). The most frequent comorbid diagnoses were affective disorders (81.0%) and other anxiety disorders (62.0%). Sixteen participants (76.0%) fulfilled the BPD diagnosis, whereas five participants (24.0%) showed a subclinical manifestation by fulfilling four criteria. Table 1 shows a detailed description of the proportion of participants fulfilling each of the BPD symptom criteria. All patients had received prior treatment; 14 (66.7%) reported prior outpatient treatment and 18 (85.7%) reported prior inpatient treatment. Seventeen patients (81.0%) received psychopharmacological medication at the beginning of the intervention and were asked not to change medication until after the follow-up assessment.

**Table 1.** Proportion of participants (*N* = 21) currently fulfilling each of the criteria for borderline personality disorder according to the International Personality Disorder Examination at pretreatment.

Borderline personality disorder criteria	Percentage of participants (*n*)
1. Frantic efforts to avoid abandonment	47.6% (10)
2. Unstable, intense interpersonal relationships	71.4% (15)
3. Identity disturbance	52.4% (11)
4. Impulsivity in at least two areas that are potentially self- damaging	38.1% (8)
5. Recurrent suicidal behaviour, threats, gestures or self-mutilating behaviour	71.4% (15)
6. Affective instability	90.5% (19)
7. Chronic feeling of emptiness	66.7% (14)
8. Inappropriate, intense anger or difficulty controlling anger	42.9% (9)
9. Transient, stress-related paranoid ideation or severe dissociative symptoms	71.4% (15)

### 3.2. Primary outcomes

Of the 21 participants, 17 completed the intervention. Four dropped out: one patient moved away; in one case, the therapeutic team ended treatment because of violations of the treatment contract (multiple relapses of alcohol use in a patient with former substance abuse disorder) and rehabilitation was recommended; and two patients left for motivational reasons. None of the patients dropped out during the exposure phase. Thus, 19% of the patients dropped out of the treatment, reflecting normal acceptance of the treatment by the patients (see Discussion). Mean treatment length was 6.6 months (range: 20–39 weeks), with an average of 21.38 sessions (range: 19–26 sessions), of which an average of 5.57 sessions (range: 4–11 sessions) included exposure elements.

Treatment safety was good. In the first phase of the treatment, one patient relapsed into alcohol dependence and was excluded from the study. According to the SBD-I, in the following, no patient showed an increase of any other problematic behaviour, such as suicidality, self-harm or need for clinical intervention. Before entering treatment, 81% of the patients reported having attempted suicide one or more times in the past, 81% of patients reported exhibiting self-injurious behaviour in the past and 52.3% reported this type of behaviour during the last four weeks. At follow-up, five of the 14 study completers (35.7%) reported to have displayed self-injurious behaviour within the last four weeks. We found a decrease in the percentage of patients showing the following self-injurious behaviours pretreatment vs. follow-up: scarifying, 9.5% vs. 7.1%; burning, 4.8% vs. 0.0%; scratching, 19.0% vs. 7.1%; manipulation of wounds, 23.8% vs. 7.1%; cutting, 28.6% vs. 21.4%; beating, 4.8% vs. 7.1%; biting, 4.8% vs. 0.0%; ripping out hair, 9.5% vs. 0.0%; other, 19.0% vs. 0.0%.

Table 2 shows the pre- to post-treatment effect sizes for study completers and ITT. The hierarchical linear model indicated a significant reduction in PTSD symptoms, as measured with the CAPS interview, regarding multiple traumas (*t*(15.12) = −5.44; *p* < .001) as well as regarding the index trauma (*t*(14.54) = −6.27; *p* < .001). The effect sizes for study completers as well as for the ITT sample from pre- to post-treatment as well as from pretreatment to follow-up were large, whereas no further improvements occurred between post-treatment and follow-up (see Table 2). At post-treatment and follow-up, 11 of the 14 study completers no longer fully met PTSD diagnostic criteria, according to the CAPS interview, so the remission rate was 79% for study completers. Regarding the ITT sample also including the study dropouts after the post-treatment assessment, 12 of the 21 patients showed remission at post-treatment, which is a remission rate of 57%.

**Table 2.** Descriptive statistics and effect sizes regarding primary and secondary outcomes for study completers (*N* = 14) and intention to treat (*N* = 21).

	Pre mean (*SD*)	Post mean (*SD*)	FU Mean (*SD*)	Pre–post effect sizes^a^ [KI]	Pre-FU effect sizes^a^ [KI]	Post-FU effect sizes^a^ [KI]
**Study completers**						
**CAPS multiple traumas**						
Total score	78.86	42.50	43.29	1.40	1.30	0
	(17.93)	(33.02)	(35.87)	[0.54;2.20]	[0.44;2.07]	[−0.76;0.72]
Intrusions	23.71	11.93	11.00	1.20	1.20	0.10
	(7.88)	(11.54)	(12.24)	[0.38–2.00]	[0.42;2.05]	[−0.66;0.82]
Avoidance	32.07	14.29	14.86	1.40	1.30	0
	(8.87)	(15.06)	(15.74)	[0.60–2.28]	[0.52;2.17]	[−0.7;0.78]
Arousal	23.07	16.29	17.43	0.80	0.60	0.10
	(7.50)	(10.13)	(11.41)	[−0.01–1.53]	[−0.17;1.34]	[−0.85;0.64]
**CAPS index trauma**						
Total score	77.79	39.71	40.57	1.40	1.30	0
	(19.90)	(32.86)	(33.65)	[0.57;2.23]	[0.52;2.17]	[−0.77;0.71]
Intrusions	23.86	10.21	9.79	1.30	1.40	0
	(8.67)	(12.13)	(11.57)	[0.47–2.11]	[0.55;2.21]	[−0.71;0.78]
Avoidance	31.21	13.21	13.36	1.50	1.40	0
	(9.03)	(15.00)	(15.07)	[0.61–2.29]	[0.60;2.27]	[−0.75;0.73]
Arousal	22.71	16.29	17.43	0.70	0.50	0.10
	(7.62)	(10.13)	(11.41)	[−0.05–1.48]	[−0.21;1.30]	[−0.85;0.64]
**DTS**						
Total score	87.50	40.14	42.86	1.50	1.50	0.10
	(24.08)	(37.67)	(35.62)	[0.65;2.34]	[0.63;2.31]	[−0.82;0.67]
Frequency	42.36	21.14	23.43	1.30	1.20	0.10
	(12.14)	(18.99)	(18.07)	[0.51–2.16]	[0.42;2.04]	[−0.87;0.62]
Severity	45.14	19.00	19.43	1.60	1.70	0
	(12.69)	(19.03)	(17.95)	[0.75–2.48]	[0.79;2.52]	[−0.76;0.72]
**IPDE**						
Dimension Current score	12.86	5.86	6.07	2.40	1.60	0
	(2.11)	(3.63)	(5.61)	[1.38;3.34]	[0.74;2.46]	[−0.79;0.70]
**BSL**						
Total score	47.29	23.50	23.71	1.10	1.20	0
	(18.69)	(25.52)	(21.12)	[0.27;1.86]	[0.37;1.99]	[−0.75;0.73]
**BDI-II** (*n* = 13^b^)						
Total score	39.85	19.76	19.82	1.50	1.50	0
	(9.58)	(16.76)	(15.26)	[0.60;2.35]	[0.68;2.46]	[−0.77;0.77]
**FDS** (*n* = 12^b^)						
Total score	48.25	24.25	20.00	0.60	0.80	0.10
	(38.25)	(43.83)	(30.99)	[−0.24;1.40]	[−0.04;1.65]	[−0.69;0.91]
**Intention to treat**						
**CAPS multiple traumas**						
Total score	79.43	49.43	50.00	1.10	1.10	0
	(16.87)	(33.04)	(34.86)	[0.49;1.80]	[0.43;1.72]	[−0.62;0.59]
Intrusions	24.38	14.52	13.62	1.00	1.00	0.10
	(7.40)	(11.67)	(12.59)	[0.37–1.65]	[0.40;1.69]	[−0.53;0.68]
Avoidance	31.76	17.67	18.05	1.20	1.10	0
	(8.17)	(14.79)	(15.18)	[0.52;1.84]	[0.47;1.78]	[−0.63;0.5 8]
Arousal	23.29	17.24	18.33	0.70	0.60	0.10
	(6.63)	(9.62)	(10.21)	[0.11–1.36]	[−0.04;1.19]	[−0.72;0.50]
**CAPS index trauma**						
Total score	78.19	47.14	48.00	1.20	1.10	0
	(17.79)	(33.53)	(33.79)	[0.50;1.81]	[0.47;1.77]	[−0.63;0.58]
Intrusions	24.19	12.95	12.67	1.10	1.10	0
	(7.87)	(12.47)	(12.33)	[0.43–1.731]	[0.46;1.77]	[−0.58;0.63]
Avoidance	30.95	16.95	16.95	1.20	1.20	0
	(8.16)	(15.00)	(15.04)	[0.50–1.82]	[0.50;1.81]	[−0.60;0.60]
Arousal	23.05	17.24	18.38	0.70	0.50	0.10
	(6.73)	(9.62)	(10.21)	[0.08;1.32]	[−0.08;1.16]	[−0.72;0.49]
**DTS**						
Total score	90.42	49.76	51.57	1.30	1.30	0
	(21.69)	(39.04)	(37.31)	[0.62;1.95]	[0.61;1.94]	[−0.82;0.67]
Frequency	43.84	25.13	26.65	1.30	1.20	0.10
	(11.37)	(18.81)	(17.89)	[0.51;2.16]	[0.42;2.04]	[−0.65;0.56]
Severity	46.58	24.63	24.91	1.30	1.30	0
	(11.09)	(20.55)	(19.82)	[0.66;2.00]	[0.68;2.02]	[−0.62;0.59]
**IPDE**						
Dimension Current score	12.81	7.57	7.81	1.40	1.10	0
	(2.42)	(4.62)	(5.70)	[0.74;2.10]	[0.49;1.80]	[−0.65;0.56]
**BSL**						
Total score	47.90	30.38	30.52	0.80	0.80	0
	(18.13)	(27.14)	(24.51)	[0.13;1.39]	[0.18;1.44]	[−0.60;0.60]
**BDI-II**						
Total score	40.05	24.53	24.88	1.10	1.10	0
	(9.41)	(17.56)	(16.66)	[0.45;1.75]	[0.47;1.77]	[−0.63;0.58]
**FDS**						
Total score	46.30	27.25	23.85	0.50	0.70	0.1
	(34.65)	(35.88)	(27.09)	[−0.08;1.16]	[0.10;1.35]	[−0.50;0.71]

CAPS = Clinician-Administered PTSD Scale; DTS = Davidson Trauma Scale; IPDE = International Personality Disorder Examination; BSL = Borderline Symptom List, BDI-II = Beck Depression Inventory; FDS = German adaptation of the Dissociative Experience Scale.

^a^indicates that effect sizes are reported as Cohen´s d.

^b^indicates that the number of patients is lower here, due to missing questionnaires at baseline.

In the hierarchical linear models regarding self-reported PTSD symptom severity, as measured with the DTS, there were also significant reductions over time (frequency: *t*(10.42) = −4.83; *p* = .001; severity: *t*(12.09) = −5.42; *p* < .001). The effect sizes for the total DTS score for study completers and ITT analyses were large (see Table 2) from pre- to post-treatment as well as from pretreatment to follow-up, and no further improvements from post-treatment to follow-up were found.

Regarding BPD symptom severity, the hierarchical linear model indicated a significant reduction over time as well, as measured by the IPDE (current dimensional score: last four weeks) (*t*(50.92) = −5.14; *p* < .001). The effect sizes for study completers and ITT from pre- to post-treatment as well as from pretreatment to follow-up were large (see Table 1). There were no changes from post-treatment to follow-up. At the post-treatment assessment, none of the 14 study completers who had met full BPD diagnostic criteria at pretreatment fulfilled the criteria regarding the past four weeks anymore, according to the IPDE interview, representing a remission rate of 100% for study completers and 67% for the ITT sample.

In the hierarchical linear models regarding self-reported BPD symptoms, as measured with the BSL, there were also significant reductions (*t*(46.40) = −3.80; *p* < .001) from pre- to post-treatment and from pretreatment to follow-up. Again, the effect sizes for study completers as well as in the ITT sample were large (see Table 2). There were no changes from post-treatment to follow-up.

### 3.3. Secondary outcome

The respective effect sizes can be found in Table 2. Secondary outcomes showed a significant reduction in depressive symptoms, as measured with the BDI (*t*(46.59) = −4.01; *p* < .001). Effect sizes for study completers and ITT analyses from pre- to post-treatment as well as from pretreatment to follow-up were large, whereas no changes occurred from post-treatment to follow-up (see Table 2). Regarding dissociative symptoms as measured with the FDS, there were also significant reductions (*t*(42.15) = −2.55; *p* = .015), with effect sizes in the medium range for study completers and the ITT sample from pre- to post-treatment and pretreatment to follow-up. There were no additional changes from post-treatment to follow-up.

## 4. Discussion

This pre–post clinical pilot trial examined the acceptance, safety and treatment effects of outpatient DBT-PTSD for women suffering from CSA-related PTSD plus symptoms of emotion regulation difficulty, treated by therapists who were novice to the treatment. Initially, patients showed severe PTSD, as reflected by an average pretest score of 79 on the CAPS severity score.

Treatment acceptance was good. At post-treatment, 19% of the patients had dropped out of treatment. However, this percentage of dropouts is comparable to that of PTSD treatments in general (18%, as reported in the meta-analysis by Imel, Laska, Jakupcak, & Simpson, 2013). Furthermore, it is lower than the dropout rate of 22.2% reported for treatment studies on CSA-related PTSD (Ehring et al., 2014) and lower than the drop-out rate of other studies focusing on patients suffering from PTSD plus BPD (Harned, Korslund, Foa, & Linehan, 2012: 23.1%; Harned, Korslund, & Linehan, 2014: 41.2%).

Treatment safety of outpatient DBT-PTSD was high: one of the patients relapsed into alcohol dependence during the first phase of the treatment. However, although all patients received trauma-focused treatment consisting of formal exposure to trauma memories, according to the SBD-I (Bohus & Borgmann, 2012), no patient showed any other form of crisis; thus, outpatient DBT-PTSD appears to be safe. The percentage of patients showing self-injurious behaviour in the past four weeks decreased from 52.3 to 35.7% from pretreatment to follow-up.

Pre- to post-treatment as well as pretreatment to follow-up effects of DBT-PTSD were found to be large for observer-rated and self-rated PTSD symptoms, borderline symptoms and depression. Effect sizes in the medium range were found for dissociation. The PTSD scores and those of secondary outcomes remained stable from post-treatment to follow-up. Changes in dissociative symptoms were smaller than those for the other primary and secondary outcomes: 97% of the patients who completed all assessments and 57% of the whole sample showed remission from PTSD. All patients who had met DSM-IV BPD criteria pretreatment and who had completed all assessments no longer fulfilled the criteria for BPD in the past four weeks at post-treatment (67% of the ITT sample).

The uncontrolled pre- to post-treatment effects sizes found in the current study are comparable to those of other trauma-focused outpatient treatments reported in a meta-analysis for CSA-related PTSD (Ehring et al., 2014), although our sample was specifically selected for more severe comorbid symptomatology in emotion regulation. One treatment that addresses similar problems for PTSD related to childhood abuse is STAIR, in which skills training in affect and interpersonal regulation precedes exposure therapy (Cloitre et al., 2010). In a randomized controlled trial addressing patients with PTSD related to childhood abuse, Cloitre et al. (2010) compared STAIR with a combination of supportive counselling and exposure therapy and found STAIR to be superior in reducing PTSD symptoms. Effect sizes on PTSD symptoms for the STAIR condition were even higher than those in our study; however, the rate of patients with BPD was lower (24 vs. 76% in our study), although it was a sample with high comorbidity and PTSD chronicity. Additionally, the dropout rate for the STAIR condition was similar to that in our study and was significantly lower than that in the support/exposure condition, which emphasizes that skills training before exposure, which is also an important part of DBT-PTSD, might be helpful to increase treatment efficacy and to reduce dropout rates in patients with PTSD and co-occurring emotion regulation difficulties.

Furthermore, in our study, pre- to post-treatment effect sizes for borderline symptoms were high, compared to those found in psychological treatment trials focusing on BPD, as summarized in a recent meta-analysis by Cristea and colleagues (Cristea et al., 2017), although BPD symptoms were only one focus of the treatment and PTSD symptoms were the main focus.

### 4.1. Limitations

The current sample is small, and the current design does not allow for the determination of whether or how the different components of the treatment contributed to the symptom improvement. The uncontrolled trial does not allow a determination of whether DBT-PTSD is superior to other, well-established treatments for PTSD in our sample. Currently, we are completing a large randomized controlled trial comparing outpatient DBT-PTSD to Cognitive Processing Therapy (CPT; Müller-Engelmann, Dittmann, Weßlau, & Steil, 2016; Resick et al., 2008) in female patients meeting the same inclusion and exclusion criteria as in the present pilot trial.

In sum, our findings indicate that outpatient DBT-PTSD is safe and has promise for reducing symptoms of PTSD and BPD as well as depression.
